# Therapeutic effect of exogenous tumor necrosis factor-stimulating protein 6 intervention on lung injury in newborn rats by intrauterine infection

**DOI:** 10.1038/s41598-025-01330-6

**Published:** 2025-05-13

**Authors:** Qing-yan Kang, Zhi-yong Sun, Xi-zhe Yuan, Zi-yang Su, Dong-yuan Xu, Wei Zhu

**Affiliations:** 1 Yanbian University Hospital, Yanji, 133099 People’s Republic of China; 2 Jilin Women and Children Health Hospital, 4 Qinghua Road, Chaoyang District, Changchun City, Jilin Province People’s Republic of China; 3 Yanbian University, Yanji, 133002 People’s Republic of China

**Keywords:** Exogenous tumor necrosis factor-stimulating protein 6, Newborn rat, Lung injury, Intrauterine infection, Diseases, Medical research, Paediatric research

## Abstract

To investigate the therapeutic effect of exogenous tumor necrosis factor-stimulating protein 6(TSG-6) on lung injury in newborn rats induced by intrauterine infection, and to analyze its underlying mechanism. Twelve pregnant rats were randomly divided into a blank control group, a negative control group, a model group, and a TSG-6 treatment group. The blank control group was not modeled. The negative control group was injected with normal saline (NS) intraperitoneally on gestation day (E)14, and the remaining two groups were intraperitoneally injected with lipopolysaccharide at 0.6 mg/kg × body weight(kg) on E14 to establish the intrauterine infection model. The negative control and model groups were injected with NS via the tail vein on E16, and the TSG-6 treatment group was injected with TSG-6 at 0.25 mg/kg × body weight(kg) via the tail vein on E16. After delivery, the placentas of pregnant rats and the right lungs of newborn rats on postnatal day (P) 3, 7, and 14 were stained with hematoxylin-eosin to observe the inflammatory infiltration of placentas, the pathology of the lungs, and the radical alveolar count (RAC) was performed. The left lung tissues of newborn rats were collected on P3, P7d, and P14. Using Enzyme-linked immunosorbent assay (ELISA) kits to measure the levels of TSG-6, tumor necrosis factor-α(TNF-α), vascular endothelial growth factor (VEGF), and Interleukin-6 (IL-6) in lung tissues of newborn rats. Compared with the model group, the growth of the control group and the TSG-6 treatment group was better, the bronchial epithelial structure of the control and TSG-6 treatment group was intact, and the epithelial cells were normal and closely arranged. The levels of RAC, VEGF, and TSG-6 in the TSG-6 treatment group were significantly increased, and the levels of IL-6 and TNF-α were significantly decreased at the early stage of life compared with the model group; the differences were statistically significant (*P* < 0.05). TSG-6 intervention can reduce the inflammatory response of pregnant rats with intrauterine infection and significantly improve the pathological degree of lung injury in newborn rats caused by intrauterine infection. Its mechanism may be related to promoting the increase of TSG-6 and VEGF levels, regulating the balance of inflammatory factors and promoting tissue repair.

**Supplementary Information:**

The online version contains supplementary material available at 10.1038/s41598-025-01330-6.

## Introduction

Intrauterine infection, as one of the common perinatal complications, may lead to fetal death, preterm labor, low birth weight, and infection. It can also lead to a series of serious sequelae, such as bronchopulmonary dysplasia (BPD), periventricular leukomalacia, and cerebral palsy, and even neonatal death^1–3^. It poses a serious threat to maternal, fetal, and neonatal health. The most common one is neonatal lung injury (LI). Its main clinical manifestations are neonatal respiratory distress, progressive dyspnea until respiratory failure, etc. The pathological process is complex and involves over-activation of inflammation, enhanced oxidative stress, and increased apoptosis, which ultimately leads to the destruction of pulmonary structure and severe impairment of pulmonary function^2,4,5^. In recent years, the mechanisms of neonatal LI due to intrauterine infections have been better understood because of the deepening of molecular biology and immunology research. Infection-induced inflammatory waterfall effects are one of the main drivers of LI. The massive release of various inflammatory cytokines, such as tumor necrosis factor-α (TNF-α) and interleukin-6 (IL-6), promotes the clearance of pathogens but also exacerbates damage to the lung tissue^6^. Enhanced oxidative stress and imbalance of antioxidant defense systems are also important factors in LI.

The current conventional treatment of intrauterine infections consists mainly of using perinatal antibiotics and glucocorticoid therapy. Perinatal antibiotic therapy includes antenatal therapy for pregnant women and empirical or therapeutic antibiotic therapy for newborns after delivery. However, there are risks associated with the use of antibiotics, such as the spread of antibiotic resistance^7^. It can also directly or indirectly alter the development of oral and intestinal microbiota in newborns^7,8^, which in turn reduces the defense response of the digestive tract, and may also increase the risk of children growth retardation, obesity, diabetes, inflammatory bowel disease, asthma, and so on^9,10^. Dysregulated microbial ecology may lead to serious adverse outcomes such as necrotic enterocolitis of newborn, sepsis, and fungal infections in newborns^7^. At the same time, prenatal dexamethasone treatment has adverse effects on the development of fetal organs and metabolic changes, and it may induce diseases in adulthood, such as hypertension, diabetes mellitus, depression, and even genetic risks^11^. Given the severity and complexity of neonatal LI due to intrauterine infections and the limitations of conventional treatment modalities, the search for effective interventions is a major focus and challenge in neonatology, especially interventional treatments targeting the pathophysiologic mechanisms of LI^12^.

Tumor necrosis factor-stimulating protein 6 (TSG-6) is the secreted protein product of TNF-stimulating gene 6. It is constitutively expressed in tissues with high basal metabolism (e.g., brain, pancreas) and in cells that provide barriers or play a role in host immune defenses (e.g., amnion, lungs, skin) normally^13^. Previous studies have shown that mesenchymal stem/stromal cells (MSCs) secrete a variety of anti-inflammatory factors, including TSG-6, through paracrine mechanisms to promote tissue repair^14–16^. As a multifunctional inflammatory factor, TSG-6 has shown great potential in recent years for research in the field of inflammation and damage repair. TSG-6 can inhibit the expression and activity of a variety of inflammatory cytokines, attenuate the inflammatory response, promote the remodeling and repair of the extracellular matrix, and accelerate the healing of tissue injury^13,17^. These properties make TSG-6 a highly promising candidate molecule for intervention in LI in newborns caused by intrauterine infection. Our study aimed to investigate the role and mechanism of TSG-6 as an intervention in the treatment of LI in newborn rats induced by intrauterine infection. The specific contents are as follows.

## Methods

### Animals

All Animal experimental procedures were approved by the Institutional Animal Care and Use Committee of The First Hospital of Jilin University (Approval No. 20230529-05), China, in compliance with the Guide for the Care and Use of Laboratory Animals published by the US National Institutes of Health (NIH publication no. 85 -23, revised 1996). All methods are reported in accordance with ARRIVE guidelines.

Animals were purchased from Beijing HFK Bioscience Co., Ltd., License No.: SCXK (Beijing)2019-0008. Eighteen 8-week-old Sprague-Dawley (SD) rats (12 females and 6 males, weighing 210–230 g) with identical birth conditions were used in this study. They were fed in 3 cages (2 cages for females and 1 cage for males, 6 rats/cage) before the start of the experiment. The rearing environment was maintained at 24 ± 1 °C, relative humidity of 40–60%, and a 12-h day/night lighting cycle. All rats were free to drink and eat under routine conditions, fed with irradiated SPF-grade sterile feed and pure water, and acclimated for 1 week before experiments. The institutional and national guide for the care and use of laboratory animals was followed.

### Reagents and instruments

TSG-6 was purchased from CUSABIO Co., USA (Catalog Number: CSB-EP023959HU, Batch number: YA04405b1g5). Lipopolysaccharide (LPS) purchased from AIBIXING Biotechnology Co., LTD., Shanghai, CN (Product number: abs47014848). The Sodium pentobarbital was purchased from New Asia Pharma Co., LTD. Shanghai, CN (Batch number: H31021725). TSG-6, TNF-α, vascular endothelial growth factor (VEGF), and IL-6 rat Enzyme-linked immunosorbent assay (ELISA) kits were purchased from Tianjin Anoric Bio-technology Co., LTD, Tianjin, CN (Catalog number: TAE-522r, TAE-138r, TAE-182r, TAE-385r). The 4% paraformaldehyde (PFA) was purchased from Biosharp Beijing Labgic Technology Co., LTD, Beijing, CN (Product number: BL539A). Hematoxylin-Eosin (H&E) staining solution was purchased from Yiyuan Biotechnology Co., LTD, Changchun, CN (Product number: Y1002). The Eclipse Ci orthogonal light microscope was purchased from Nikon Co., Japan (Model number: Nikon Eclipse CI), etc.

### Grouping and modeling

**Fig. 1:**
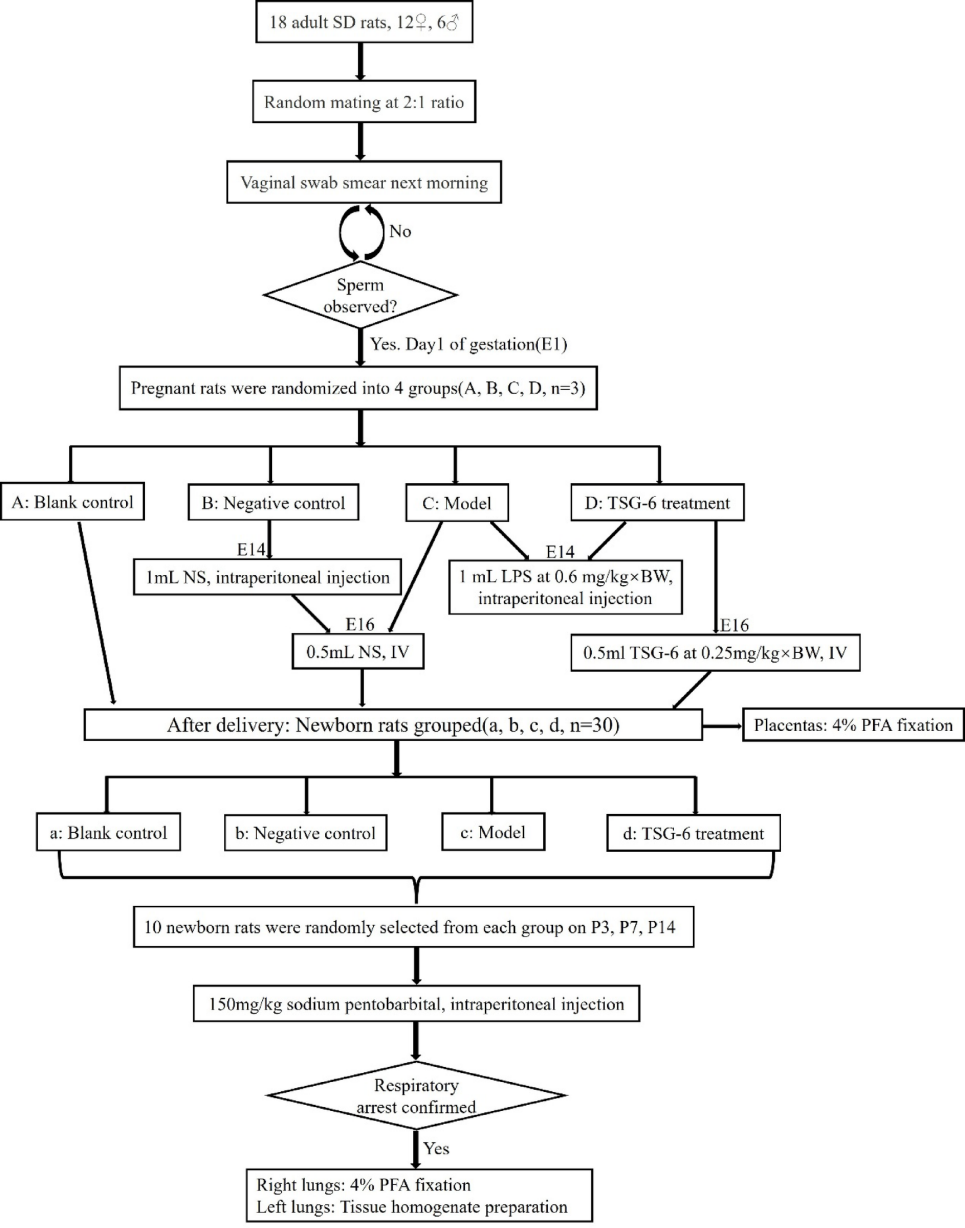
Study design and experimental grouping.

Eighteen adult SD rats (12 females and 6 males) were randomly mated and impregnated according to a 2:1 ratio. A vaginal swab smear was done the next morning, and when sperm was seen under the microscope, it was the first day of gestation(E1). Pregnant rats were randomly divided into a blank control group (group A), a negative control group (group B), a model group (group C), and a TSG-6 treatment group (group D), with 3 rats in each group. Newborn rats were obtained from each group after delivery (groups a–d, 30 newborn rats in each group). Group B was injected with 1 mL of normal saline (NS) intraperitoneally on E14. Groups C and D were intraperitoneally injected with 1 mL of LPS at 0.6 mg/kg × body weight(kg) on E14. Group B and C were injected with 0.5 mL of NS via the tail vein on E16, and Group D was injected with 0.5 mL of TSG-6 at 0.25 mg/kg × body weight(kg) via the tail vein on E16. After delivery, the placentas of pregnant rats were fixed in 4% PFA. On the third postnatal days(P), P7, and P14, 10 newborn rats were randomly selected from each group and euthanized by intraperitoneal injection of 150 mg/kg sodium pentobarbital. Respiratory arrest was used as an indicator of completion of euthanasia. The right lungs were fixed in 4% PFA, and the left lungs were prepared as tissue homogenates and stored for examination (Fig. 1: Study design and experimental grouping).

### Experimental evaluation

#### H&E Staining

The 4% PFA-fixed placental and right lung tissue samples were dehydrated, embedded, sectioned, stained, and sealed to make pathological sections. The samples were observed under the microscope and photographed. Describing the basic pathological changes in the pathological sections, such as congestion, hemorrhage, bruising, edema, degeneration, necrosis, hyperplasia, fibrosis, and inflammatory cell infiltration.

#### Radical alveolar count (RAC)

Sections of the above lung tissues were observed under a 100× light microscope. A vertical line was drawn from the center of the respiratory fine bronchioles to the nearest fibrous septum or pleura, and the number of alveoli on that line was counted. Three sections of each rat lung tissue were taken for observation, and five fields of view were randomly selected from each section, and then the mean value was calculated.

#### Detection of cytokines in lung tissue

Lung tissue homogenates were prepared from the left lungs of newborn rats on P3, P7, and P14. Add 5 times the volume of homogenization medium (phosphate buffer saline solution, PBS) at the ratio of weight (mg): volume (µL) = 1:5, and mechanically homogenized under the condition of ice-water bath to prepare 20% homogenate, centrifuge for 10 min at 2500–3500 rpm at 4 °C, and then extract the supernatant. Using ELISA to measure the levels of IL-6, TNF-α, VEGF, and TSG-6 in lung tissues of newborn rats, the specific assays were performed according to the instructions of the corresponding kits.

### Statistical analysis

Data were processed and analyzed using SPSS 26.0 and GraphPad Prism software 10.0 for Windows. Measurement data were expressed as mean ± standard deviation (${\bar{\text{x}}}$ ± s) and interquartile range. Choosing one-way analysis of variance (ANOVA) or nonparametric tests, respectively, according to whether the normal distribution and homoscedasticity were satisfied. Statistical significance was defined as *P* < 0.05.

## Results

### Comparison of histopathologic changes in the placenta of pregnant rats

**Fig. 2:**
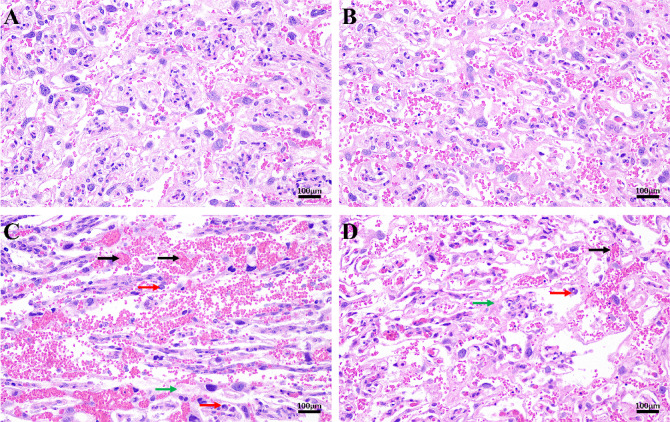
Pathology of the placenta(H&E, 200×); (**A**) Blank control group, (**B**) Negative control group, (**C**) Model group, (**D**) TSG-6 treatment group. The placental tissues in groups C and D were congested. Some interstitial edema and massive neutrophil infiltration were seen. Compared with group C, the degree of inflammatory cell infiltration was milder in group D. No difference was seen between groups A and B.

In groups C and D, the placental tissue was congested, and some interstitial edema and massive neutrophil infiltration were seen. The placental tissue structures of groups A and B were clear, with no interstitial edema or neutrophil aggregation. Compared with group C, the degree of inflammatory cell infiltration was milder in group D. No difference was seen between groups A and B. (Fig. 2: Pathology of the placenta).

### The General condition of newborn rats and comparison of body weights at different periods

**Fig. 3:**
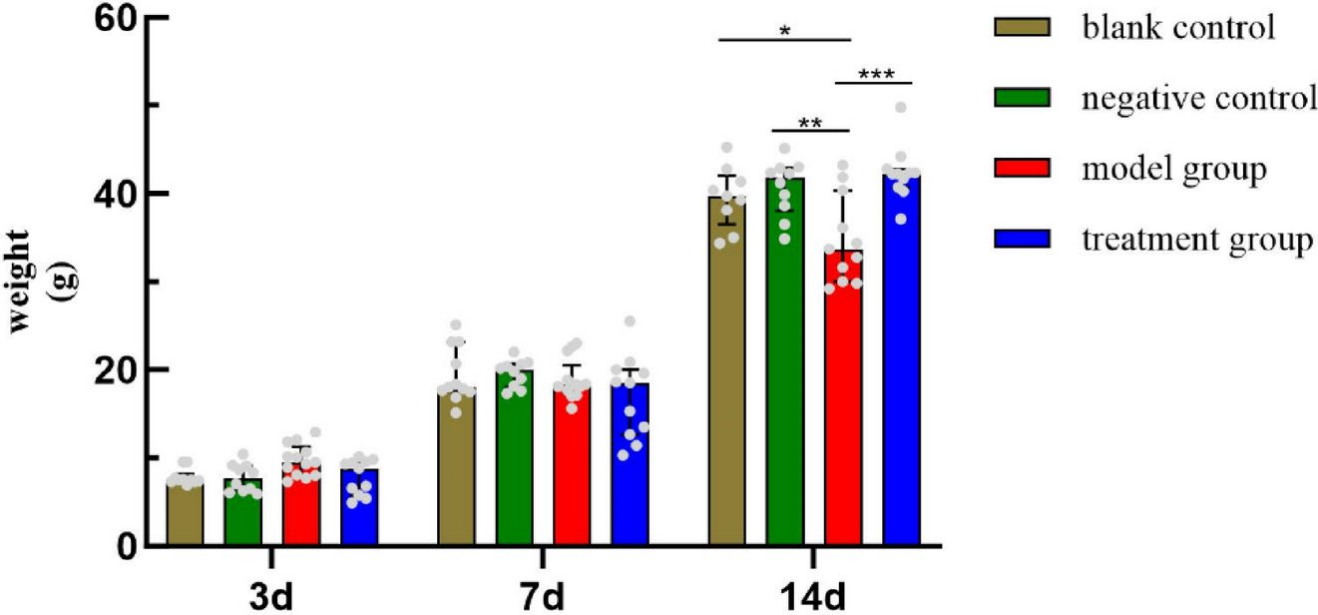
Body weight in newborn rats at different periods. On P14, the weight of group c was significantly lower compared with groups a and b. The weight of group d was significantly higher compared with group c. *P < 0.05, **P < 0.01, ***P < 0.001.

The newborn rats in each group had rosy skin and good activity on P1, and were sensitive to external stimuli, with no obvious behavioral differences. On P14, compared with groups a and b, the weight of group c was significantly lower, and compared with group c, the weight of group d was significantly higher. The difference was statistically significant (*P* < 0.05). There was no statistically significant difference (*P* > 0.05) in the comparison of body weights on P3 and P7. (Fig. 3: Body weight in newborn rats at different periods).

### Comparison of histopathologic changes in the lungs of newborn rats

**Fig. 4:**
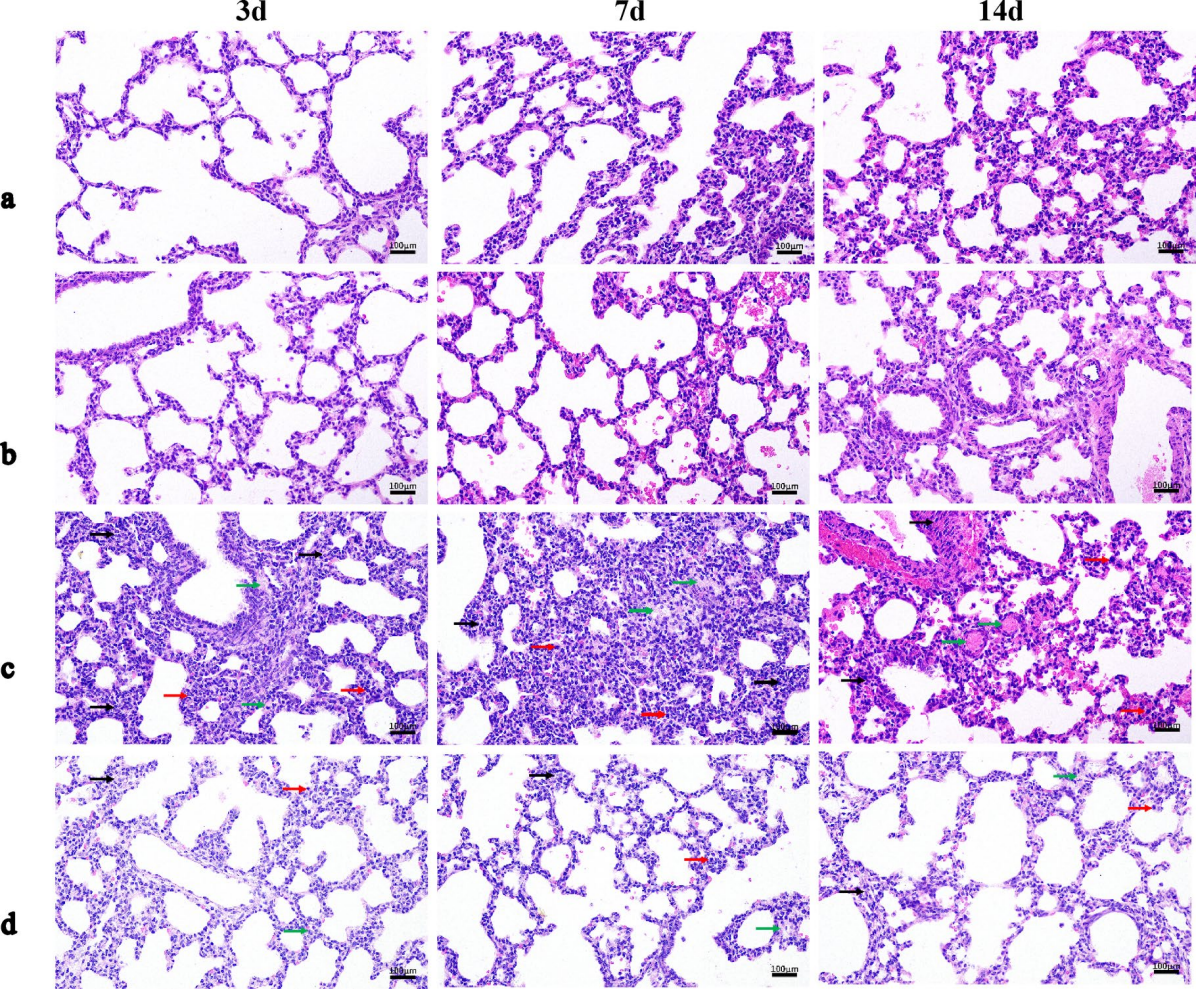
Pulmonary pathology at different periods (H&E, 200×); (**a**) Blank control group, (**b**) Negative control group, (**c**) Model group, (**d**) TSG-6 treatment group. In group c, the bronchial epithelial structure was disorganized, the alveolar wall was thickened, and more lymphocytes and macrophages could be seen. Pulmonary edema could be seen at the edge of the tissue, eosinophilic plasma-like material could be seen in some of the alveolar lumens, and thrombus could be seen in the capillaries of the local alveolar wall. In group d, the bronchial epithelial structure was intact, the epithelial cells had normal morphology and structure and were tightly arranged, the alveolar structure was intact, the local alveolar wall was slightly thickened, and a small number of lymphocytes and macrophages could be seen, and the degree of interstitial edema in the lungs was reduced compared with group c.

The bronchial epitheliums of groups a and b were intact, with normal morphology and structure of epithelial cells, tight arrangement, clear alveolar structure, no obvious thickening of alveolar wall, and a small amount of lymphocyte and macrophage infiltration was seen. In group c, the bronchial epithelial structure was disorganized, the alveolar wall was thickened, and more lymphocytes and macrophages could be seen. Pulmonary edema could be seen at the edge of the tissue, eosinophilic plasma-like material could be seen in some of the alveolar lumens, and thrombus could be seen in the capillaries of the local alveolar wall. In group d, the bronchial epithelial structure was intact, the epithelial cells had normal morphology and structure and were tightly arranged, the alveolar structure was intact, the local alveolar wall was slightly thickened, and a small number of lymphocytes and macrophages could be seen, and the degree of interstitial edema in the lungs was reduced (Fig. 4: Pulmonary pathology at different periods).

### Comparison of RAC in newborn rats

Compared with groups a and b, group c showed a significant decrease in RAC. Compared with group c, group d showed a significant increase in RAC. The difference was statistically significant (*P* < 0.05). Comparisons between groups a, b, and d for each birth period were not statistically significant(*P* > 0.05) (Table 1).

**Table 1:** RAC in newborn rats at different periods (median(P25, P75)).

	3d	7d	14d
Group a(*N* = 10)	15.01(11.77,18.03)*	16.76(16.04,18.13)*	15.48(14.96,16.63)*
Group b(*N* = 10)	14.74(13.31,19.69)*	18.09(15.70,19.75)*	15.22(14.67,15.86)*
Group c(*N* = 10)	11.39(7.70, 12.39)	8.73(7.34,10.59)	9.99(8.76,10.70)
Group d(*N* = 10)	16.00(11.39,18.44)*	16.26(14.80,17.38)*	14.45(13.26,16.79)*
H	13.147	23.955	23.375
P	< 0.05	< 0.01	< 0.01

*Indicates that compared with group c, the difference was statistically significant (*P* < 0.05). All comparisons were corrected by Bonferroni correction.

### Comparison of cytokine levels in lung tissue homogenates from newborn rats

**Fig. 5:**
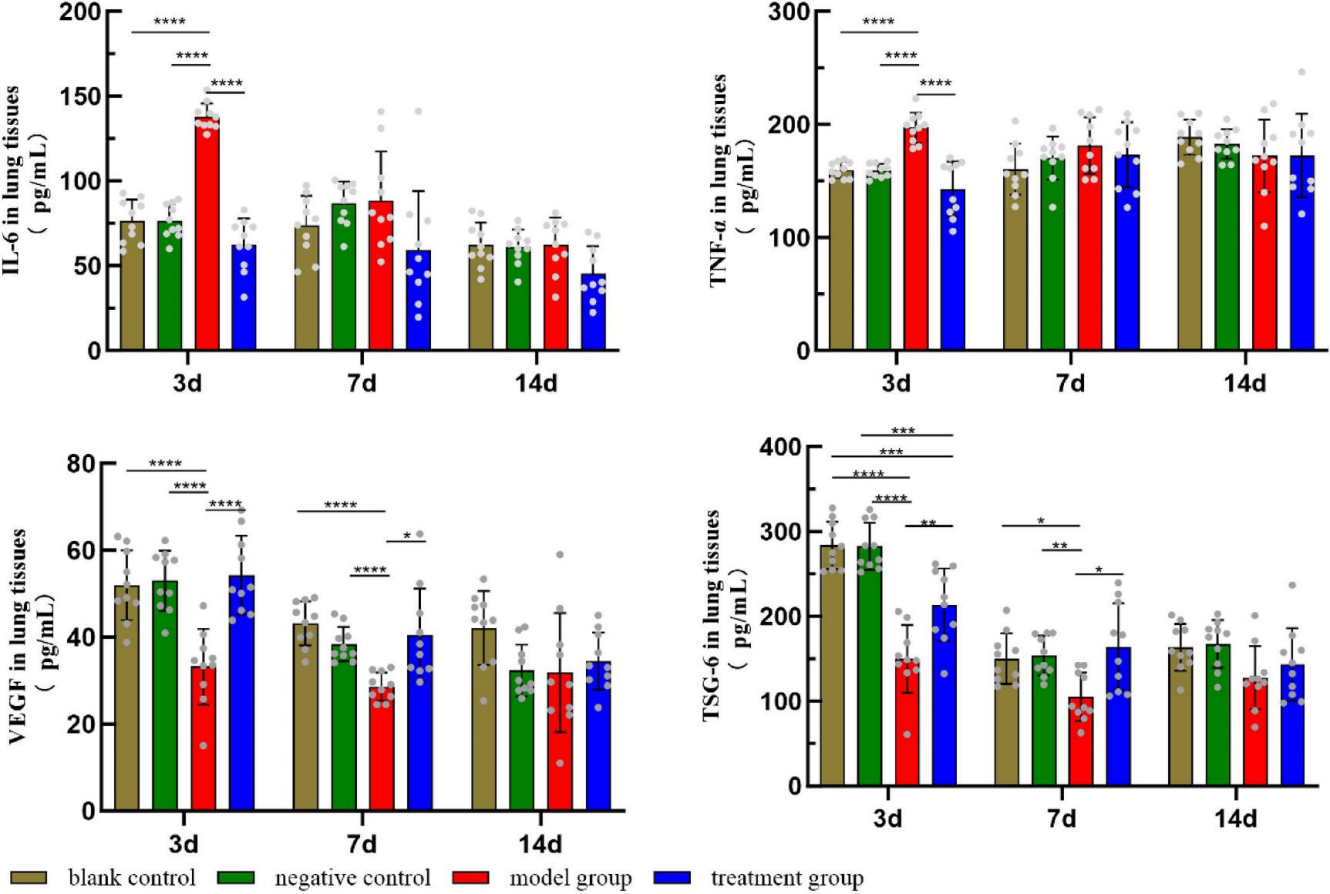
Cytokine levels in lung tissue homogenates from newborn rats. On P3: Compared with groups a and b, group c showed significantly higher levels of IL-6 and TNF-α and lower levels of VEGF and TSG-6, and group d showed significantly lower levels of TSG-6. On P7: Compared with groups a and b, the levels of VEGF and TSG-6 were significantly lower in group c. Compared with group c, the levels of VEGF and TSG-6 were significantly higher in group d. *P < 0.05, **P < 0.01, ***P < 0.001, ****P < 0.0001.

On P3: Compared with groups a and b, group c showed significantly higher levels of IL-6 and TNF-α and lower levels of VEGF and TSG-6, and group d showed significantly lower levels of TSG-6. The difference was statistically significant (*P* < 0.05). On P7: The levels of VEGF and TSG-6 were significantly lower in group c compared with groups a and b. Compared with group c, the levels of VEGF and TSG-6 were significantly higher in group d. The difference was statistically significant (*P* < 0.05). In the other periods, there was no statistically significant difference in the comparison between each group (*P* > 0.05) (Fig. 5: Cytokine levels in lung tissue homogenates from newborn rats).

## Discussion

The pathophysiologic mechanism of neonatal LI and acute respiratory distress syndrome, triggered by intrauterine infection, is increased permeability of alveolar capillaries to fluids, proteins, and inflammatory cells, resulting in enhanced inflammation, accelerated autophagy, epithelial-mesenchymal transition (EMT), and decreased alveolar fluid clearance, leading to pulmonary edema, impaired gas exchange, hypoxemia, and pulmonary fibrosis^12^. Previous studies have shown that the main cause of LPS-induced inflammation is the activation of the nuclear factor kappa-light-chain-enhancer of activated B cells (NF-κB) signaling pathway. The upregulation of this pathway will induce IL-6, IL-8, and other inflammatory factors, which in turn act on monocyte macrophages and neutrophils, causing a vicious cycle of inflammatory response^12,18^. Several studies have shown that TSG-6 can reduce the expression of pro-inflammatory factors by inhibiting the NF-κB signaling pathway^17–20^. The administration of exogenous TSG-6 via endotracheal drip significantly improved pulmonary vascular remodeling and pulmonary hypertension, while also reducing the expression of the NF-κβ pathway and inflammatory factors in rats with BPD^21^. TSG-6 can induce microglia to switch to the M2 phenotype (anti-inflammatory phenotype) for reducing neuroinflammation in rats caused by LPS^22^. Knockdown of TSG-6 in MSCs-exosome (MSCs-Exos) conditioned medium significantly reduces its anti-inflammatory therapeutic effects^16^. It can be speculated that TSG-6, which is produced by MSCs in response to inflammatory signals^23^, is an important factor that exerts immunomodulatory and reparative effects, at least in part.

In this study, a newborn rat model of LI was prepared by inducing intrauterine infection in pregnant rats by LPS. Comparing the placental pathology in all groups of pregnant rats, inflammatory cell infiltration could be seen in the placental pathology of both the model group and the TSG-6 treatment group, which indicated that our intrauterine infection model of pregnant rats was successful. Compared with the model group, the placental pathology was improved in the TSG-6 treatment group, suggesting that TSG-6 could reduce the degree of placental pathology and inflammatory reaction in intrauterine-infected pregnant rats. On P14, the body weight of the model group was lower than that of the control group, and significantly lower than that of the TSG-6 treatment group, suggesting that infection in the dams affected the normal growth of newborn rats and that TSG-6 had a positive effect on the growth of intrauterine-infected newborn rats. Comparing the lung pathology and RAC of newborn rats, the lung tissue of the model group showed thickening of alveolar walls, widening of alveolar septums, and a large number of inflammatory cells in the interstitium and alveolar lumen. Compared with the model group, the histopathology of the lungs in the TSG-6 treatment group showed that the alveolar structure was relatively intact, the alveolar septum was not significantly widened, the degree of interstitial edema was reduced, the number of inflammatory cells was significantly reduced, and RAC was significantly increased. These positive morphological changes suggest that TSG-6 may attenuate the lung tissue damage caused by intrauterine infection in newborn rats through its anti-inflammatory, anti-injury, and reparative mechanisms of action.

Under normal conditions, TSG-6 is constitutively expressed in the lungs and is also induced to be secreted when the lungs are stimulated by pro-inflammatory factors such as TNF-α and interferon-γ^13,23,24^. Previous studies have shown that in many animal models of inflammation (including keratitis, arthritis, peritonitis, BPD, etc.), the expression level of TSG-6 is elevated^16,22,23,25^. However, in our study, at the early stage of birth of the newborn rats, the TSG-6 levels in both the model group and the treatment group were lower than those in the control groups, which was not consistent with previous studies, and the TSG-6 levels in the treatment group were elevated in comparison with those in the model group. We hypothesized that TSG-6 might be in a state of rapid progressive depletion in the anti-inflammatory response in vivo. In the early stage of infection, the body may accelerated the degradation of TSG-6 through the activation of specific proteases, resulting in significantly lower TSG-6 levels in the model group and the treatment group compared with the control group, while the treatment group obtained exogenous TSG-6 supplemented from dams during the fetal period, which could temporarily restore the anti-inflammatory efficacy of the period, and thus the TSG-6 levels in the treatment group were higher than those in the model group. This phenomenon suggests that there may be a negative feedback regulatory mechanism affecting TSG-6 secretion in vivo. With the development of inflammation, the depletion and production of TSG-6 in vivo, and the gradual refinement of immune function during the growth and development of newborn rats, TSG-6 may reach a dynamic equilibrium state with related inflammatory factors. In the future, we need to continue to study the half-life of exogenous TSG-6 protein in vivo, the optimal concentration of action, and the presence of dose dependency and side effects.

VEGF, a multifunctional cell growth factor belonging to the platelet-derived growth factor family, stimulates mitosis and angiogenesis in vascular endothelial cells and increases the permeability of the endothelial monolayer. It plays a dual role in LI. In the early stage of LI, damage to alveolar epithelial cells and vascular endothelial cells reduces the production of VEGF, and the release of VEGF protease from inflammatory cells infiltrating lung tissue leads to a further reduction in the concentration of VEGF in the lungs. In the late stage of LI, alveolar epithelial cells repair and proliferate, and the concentration of VEGF in the lung gradually recovers^26,27^. There have been some studies showing that MSCs depend on TSG-6 gene expression to regulate VEGF levels in a model of corneal inflammation, which in turn inhibits inflammatory neoangiogenesis. There may be some interaction between TSG-6 and VEGF^28^. In this study, the concentration of inflammatory factors in lung tissues was measured by using ELISA. The results showed that, at the early stage of life, compared with the model group, the concentrations of TNF-α and IL-6 were significantly decreased, and the concentrations of TSG-6 and VEGF were significantly increased in the lung tissues of the TSG-6 treatment group. On P7, compared with control groups, the levels of VEGF and TSG-6 in the model group were significantly lower, and no significant differences were observed in the levels of IL-6 and TNF-α among the other groups. These changes suggest that TSG-6 may act at the early stage of inflammation to form a microenvironment conducive to lung tissue repair by inhibiting the production of key pro-inflammatory factors and up-regulating the expression of anti-inflammatory and pro-repair factors.

The limitations of this study include the following. First, there was a certain abortion rate and mortality rate during the modeling process of intrauterine-infected pregnant rats, and the female rats’ estrous cycle was not consistent, resulting in the period of modeling and delivery being separated by several days. Second, the sample size of pregnant rats was relatively small, and the drug concentration gradient was not set. Because our study was the first attempt at a new treatment modality for intrauterine infections, and based on the effectiveness of the intervention at this stage, we can further expand the sample size in the future and study whether there is a dose dependence. Finally, our study suggested an anti-inflammatory effect of TSG-6, but the molecular mechanism has not yet been clarified. This requires further research.

In conclusion, interventional treatment of intrauterine-infected pregnant rats with exogenous TSG-6 can effectively reduce the degree of LI pathology in newborn rats caused by intrauterine infection and may play a role in regulating the level of inflammatory factors and promoting tissue repair in the early stage of inflammation. Application of TSG-6 instead of, or in conjunction with, antibiotics or glucocorticoids for treating intrauterine infections will hopefully reduce the incidence of adverse maternal-fetal events, spare the newborn from empiric antibiotic therapy, and improve neonatal health. In the future, we need to further improve the experiments and deeply explore the specific molecular mechanism of TSG-6 in LI repair to provide more solid evidence for its application in clinical treatment.

## Electronic supplementary material

Below is the link to the electronic supplementary material.

Supplementary Material 1

Supplementary Material 2

Supplementary Material 3

Supplementary Material 4

Supplementary Material 5

## Data Availability

All data generated or analysed during this study are included in this published article (and its Supplementary Information files).
